# Potential role of standardized orbital ultrasound in the management of spontaneous CSF leaks

**DOI:** 10.1007/s10143-026-04277-y

**Published:** 2026-04-11

**Authors:** Mario Rigante, Carmela Grazia Caputo, Maria Grazia De Antoniis, Ludovico Agostini, Pier Paolo Mattogno, Claudio Parrilla, Liverana Lauretti, Vittorio Orlando, Stanislao Rizzo, Francesco Doglietto, Jacopo Galli

**Affiliations:** 1 Department of Otorhinolaryngology and Head-Neck Surgery, Fondazione Policlinico Universitario Agostino Gemelli IRCCS, Rome, Italy; 2 Department of Neurosurgery, Fondazione Policlinico Universitario Agostino Gemelli IRCCS, Rome, Italy; 3 Department of Ophthalmology, Fondazione Policlinico Universitario Agostino Gemelli IRCCS, Rome, Italy

**Keywords:** Spontaneous cerebrospinal fluid leak, Idiopathic intracranial hypertension, Orbital ultrasound, Optic nerve sheath diameter, Dilated optic nerve sheath

## Abstract

Spontaneous Cerebrospinal Fluid (sCSF) leak is a rare condition commonly associated with Idiopathic Intracranial Hypertension (IIH). Orbital ultrasound is an effective and non-invasive method for monitoring intracranial pressure (ICP) variations in sCSF leak patients undergoing surgical repair. This study aims to consolidate existing data by sharing our single-center, long-lasting clinical experience with Standardized Orbital Ultrasound (SOUS) evaluation in this patient cohort. Data from sCSF leak patients who underwent endoscopic surgical repair from 2003 to 2023 at Fondazione Policlinico Universitario Agostino Gemelli IRCCS (Rome, Italy) were retrospectively collected. As part of our diagnostic, therapeutic flow chart, all patients underwent a comprehensive preoperative assessment, including a head CT scan and/or brain MRI. Postoperatively, all patients were prescribed Acetazolamide. Patients with radiological findings suggestive of IIH were further evaluated with SOUS preoperatively (T0). Ultrasonographic measurements were also recorded at 3 (T1), 6 (T2), and 12 (T3) months after surgical treatment and the initiation of medical therapy. 23 out of 56 patients underwent SOUS examination preoperatively and postoperatively. The mean preoperative optic nerve sheath diameter (ONSD) was 4.8 mm ± 0.1. Postoperatively, the mean ONSD was 5.1 mm ± 0.1 at T1, 4.9 mm ± 0.2 at T2, and 4.8 mm ± 0.2 at T3. None of those patients experienced CSF leak recurrence within the first year. SOUS has shown a promising role in evaluating the quality of the surgical repair and monitoring the ICP response to Acetazolamide, thereby reducing the risk of CSF leak recurrence after surgical treatment.

## Introduction

Spontaneous Cerebrospinal Fluid (sCSF) leak is a rare condition that typically presents as rhinoliquorrhea without any identifiable cause, such as trauma or neoplastic diseases. The most involved sites are the ethmoid roof and the lateral wall of the sphenoid bone, although the leak can occur at multiple sites on the skull base [1–4]. The pathway connecting the external environment to the central nervous system (CNS) exposes the patient to severe complications, including meningitis.

Spontaneous CSF leaks can be challenging to treat effectively, as they are associated with a high risk of recurrence^,^ with success rates of primary surgery varying from 25% to 87% [2, 5–7]. The surgical management of sCSF leaks has evolved significantly, with endoscopic endonasal surgery now considered the gold standard for non-conservative treatment [3, 7–9]. Several closure techniques have been shown to ensure an optimal surgical repair, depending on the defect size, location, and associated conditions, such as meningocele or meningoencephalocele [9–20].

Several studies have highlighted a strong association between spontaneous CSF leaks and Idiopathic Intracranial Hypertension (IIH) [1, 3, 18, 19, 21–24]. Factors such as female gender, obesity, and age in the fifth to sixth decade of life are commonly associated with both conditions [3, 18]. The treatment of IIH is primarily conservative, with weight loss being of paramount importance, along with medical therapy (Acetazolamide 500–4000 mg/day) [7, 25]. The persistence of underlying intracranial hypertension after the leak has been surgically repaired is considered the primary risk factor for CSF leak recurrence [26, 27]. 

Standardized orbital ultrasound (SOUS), pioneered and developed by Karl Ossoinig, is a state-of-the-art method of diagnostic ophthalmic ultrasonography [28–31]. SOUS has emerged as a valuable, non-invasive, and reliable tool for indirect ICP monitoring: by measuring the diameter of the optic nerve sheath (ONSD), SOUS can predict elevated ICP values [30, 32–43]. A diameter of 5 mm or more is commonly considered pathognomonic for intracranial hypertension [33, 35–40, 43–46]. 

Recently, Tilak et al. [38] have demonstrated the effectiveness of standardized OUS in monitoring ICP in patients suffering from sCSF leaks despite the limited duration of follow-up.

This study aims to provide further evidence of the efficacy of standardized OUS in detecting ICP variations in patients undergoing surgical skull base repair for sCSF leaks by sharing a 20-year experience with standardized OUS in the diagnostic work-up and postoperative follow-up of sCSF leak patients.

## Materials and methods

We retrospectively collected data from 504 patients diagnosed with CSF leak at Fondazione Policlinico Universitario A. Gemelli IRCCS (Rome, Italy) from October 2003 to January 2023.

Informed consent was obtained from all individual participants included in the study. Every diagnostic and therapeutic procedure was carried out following the standards of the Lazio Area 3 Territorial Ethics Committee and the Helsinki Declaration [47].

Clinical trial number: not applicable.

Ethics approval for this study was obtained from the Lazio Area 3 Territorial Ethics Committee. Every diagnostic and therapeutic procedure was carried out in accordance with the standards of the Lazio Area 3 Territorial Ethics Committee and the Helsinki Declaration.

A total of 48 patients, with complete retrospective data collection, met the inclusion criteria for the study (Table 1).

**Table 1:** Inclusion and exclusion criteria

Inclusion Criteria	Exclusion Criteria
1. First diagnosis of spontaneous CSF leak 2. Surgical treatment at our Center 3. Age > 18 years 4. Available radiological imaging 5. Complete preoperative assessment 6. Complete OUS measurements 7. Standardized OUS protocol 8. Single expert examiner 9. Complete follow-up up (at 3, 6, and 12 months after surgery)	1. Alternative etiology for CSF leak 2. Recurrence 2. Surgical treatment outside our Center 3. Age < 18 years 4. Incomplete or absent radiological imaging 5. Absence of $\:\beta\:$-trace protein test 6. Incomplete follow-up

**Fig. 1:**
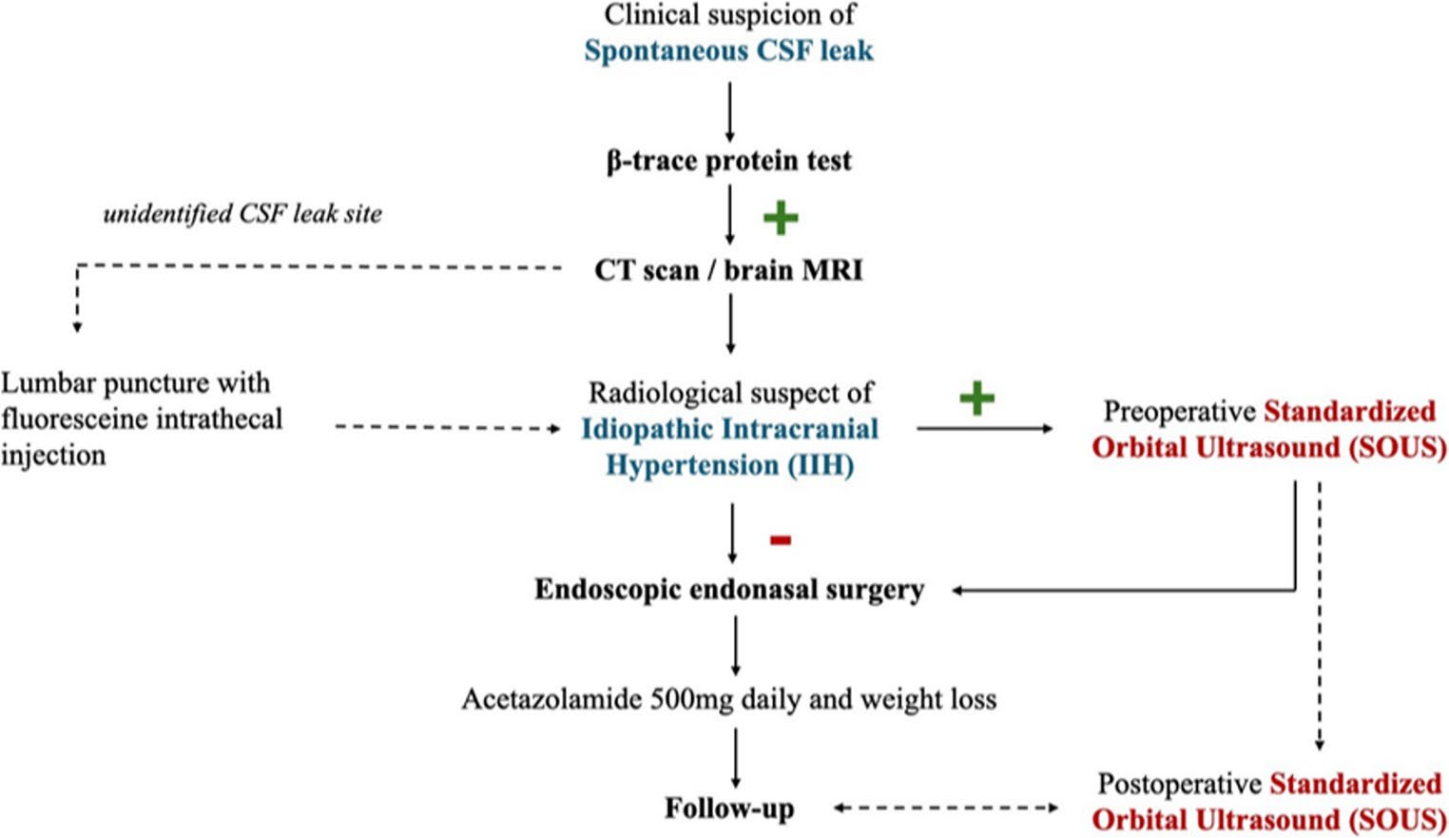
Diagnostic and therapeutic algorithm for sCSF leak at our center using standardized orbital ultrasound

Figure 1 illustrates the diagnostic-therapeutic algorithm followed at our Institution in case of a suspected CSF leak. All patients with a clinical suspicion of rhinoliquorrhea were tested for 𝛃-trace protein levels in nasal secretions. Patients who tested positive for 𝛃-trace protein underwent a high-resolution CT scan and/or an MRI with gadolinium enhancement of the head and neck to identify the site of the leak and exclude any underlying causes, such as intra- or extracranial masses. Patients without imaging to clarify the site of the leak underwent lumbar puncture with intrathecal injection of fluorescein to detect it intraoperatively.

Preoperatively, patients with MRI findings suggestive of IIH (*n* = 23/48, 48%) were further evaluated with standardized orbital ultrasound (SOUS) to assess for papilledema and measure the transverse diameter of the optic nerve sheath (ONSD).

Following surgical repair, all patients were advised to lose weight and initiate daily administration of 500 mg of Acetazolamide. Follow-up assessments were conducted at 3, 6, and 12 months. SOUS was included in the follow-up for patients with MRI findings suggestive of IIH who had also undergone preoperative OUS evaluation. No dose adjustments were made before the first postoperative follow-up visit at 3 months (T1), unless intolerable side effects required discontinuation. The decision to increase the Acetazolamide dose at T1 or later was guided by two criteria, applied individually or in combination: (1) persistence or recurrence of IIH-related symptoms (headache, visual disturbances, or pulsatile tinnitus) and/or (2) ONSD values remaining at or above the pathological threshold (5 mm) on two consecutive follow-up measurements despite ongoing therapy.

### Standardized orbital ultrasound

**Fig. 2:**
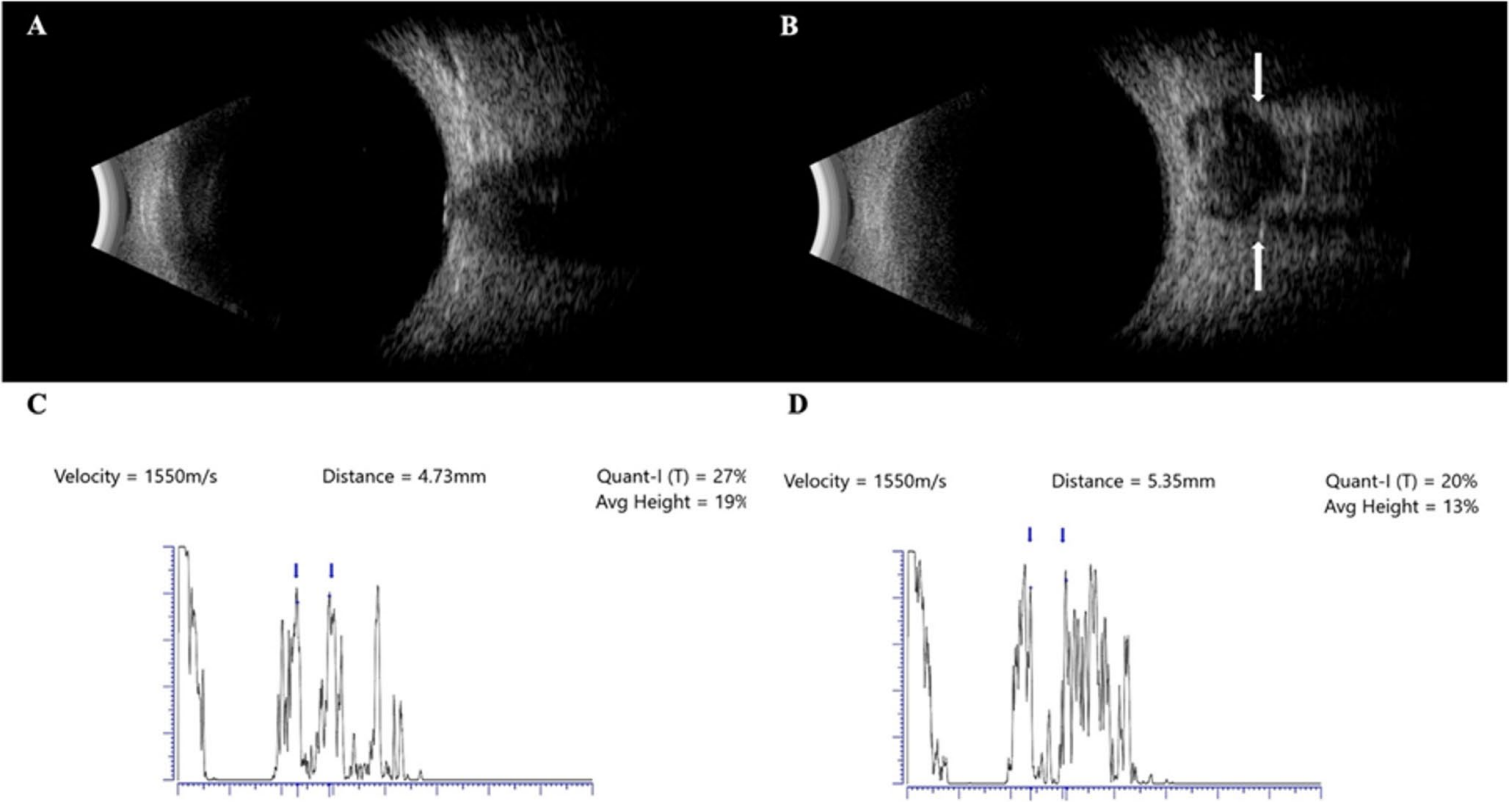
Standardized orbital ultrasound (SOUS) in a patient with spontaneous CSF leak. B-scan showing normal optic nerve preoperatively (**A**); A-scan showing normal optic nerve sheath diameter (ONSD) preoperatively (**C**), (**B**) “crescent sign” due to the subarachnoid liquid (arrow) between the optic nerve parenchyma and perineural sheath, indicative of intracranial hypertension postoperatively and abnormal ONSD after surgical repair of the fistula (**D**)

Standardized echography of the optic nerve was performed using both standard A-scan (8 MHz; Aviso S, Quantel Medical, MN, U.S.A.) and B-scan (10 MHz; Aviso S, Quantel Medical, MN, U.S.A.) (Fig. 2) [28]. All measurements were conducted by a single expert (CGC).

### Statistical analyses

The Kolmogorov–Smirnov test was preliminarily run to assess the normality of the sample distribution and the subsequent choice of the appropriate statistical test. Clinical data were analyzed using a T-Test (GraphPad, San Diego, CA, USA). The significance limit was set at *p* < 0.05 (95% confidence interval). For continuous normally distributed data, the mean ± standard deviation was used.

## Results

The following demographic data were observed, as presented in Table 2.

**Table 2:** Demographic data

Total patients	*N* ^O^
	48 (100%)
Female patients	32 (67%)
Mean age (years)	53.5 (range: 20–78)
mean BMI kg/m² (SD)	29.2 (± 7)
Patients with BMI ≥ 30 kg/m²	11 (23%)

All patients (*n* = 48/48, 100%) presented with monolateral rhinorrhoea. The main associated symptoms were headache, nausea, and vomiting. Notably, 3/48 patients (6.5%) presented with pneumococcal meningitis.

All patients (*n* = 48/48, 100%) tested positive for $\:\beta\:$-trace protein in nasal secretions.

A single fistula site was identified in most patients (*n* = 43/48, 89.5%). The main sites of leaks are reported in Table 3. In 5/48 (10.5%) cases, multiple leakage points were identified.

**Table 3:** Single sites of sCSF leak observed in our cohort of patients

Sites	*N* ^O^
Planum ethmoidalis	10
Sphenoid sinus	8
Cribriform plate	8
Fovea ethmoidalis	6
Ethmoid sinuses	5
Lateral recess of sphenoid sinus	5
Other	1

All patients underwent either a CT or an MRI scan. In 23 out of 48 patients (48%), findings consistent with IIH were observed (Table 4). These patients were further evaluated with OUS in the preoperative and postoperative periods (Fig. 1).

**Table 4:** Idiopathic intracranial hypertension (IIH) CT/MRI findings observed in our cohort of patients

Findings	*N* ^O^
Empty sella	15
Distention of perioptic subarachnoid space	8
Enlarged Meckel’s cave	6
Partially empty sella	7
Optic nerve protrusion	1
Flattening of the posterior globe	1

All patients underwent an endoscopic endonasal approach for the surgical repair of the leak. In 3 cases (*n* = 3/48, 6%), a combined approach (endoscopic endonasal and craniotomic) was required. Intraoperative fluorescein was utilized in a few cases (*n* = 11/48, 23%) when preoperative imaging did not identify the leakage site. No patient required a lumbar drain. No major complication was reported in the perioperative period. All patients (*n* = 48/48, 100%) were postoperatively prescribed diuretic therapy (Acetazolamide 500 mg/day).

**Fig. 3:**
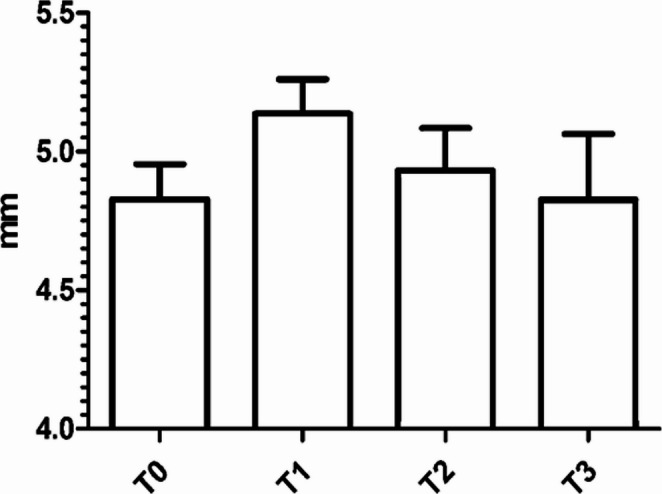
Bar plot showing mean optic nerve sheath diameters (ONSD) assessed through standardized orbital ultrasound (SOUS). Measurements were taken both preoperatively (T0) and postoperatively, at 3 (T1), 6 (T2), and 12 months (T3) after surgery. Error bars express standard deviation (SD)

The optic nerve sheath’s mean preoperative (T0) diameter was 4.8 mm ± 0.1 (Fig. 3). Three months postoperatively (T1), the mean ONSD was 5.1 mm ± 0.1 (Fig. 3). The mean difference in OUSD between T0 and T1 was + 0.3 mm ± 0.2 (*p* = 0.04, 95% CI= −0.6605 to 0.04158). Six months postoperatively (T2), the mean ONSD was 4.9 mm ± 0.2 (Fig. 3). Twelve months postoperatively (T3), the mean ONSD was 4.8 mm ± 0.2 (Fig. 3).

**Fig. 4:**
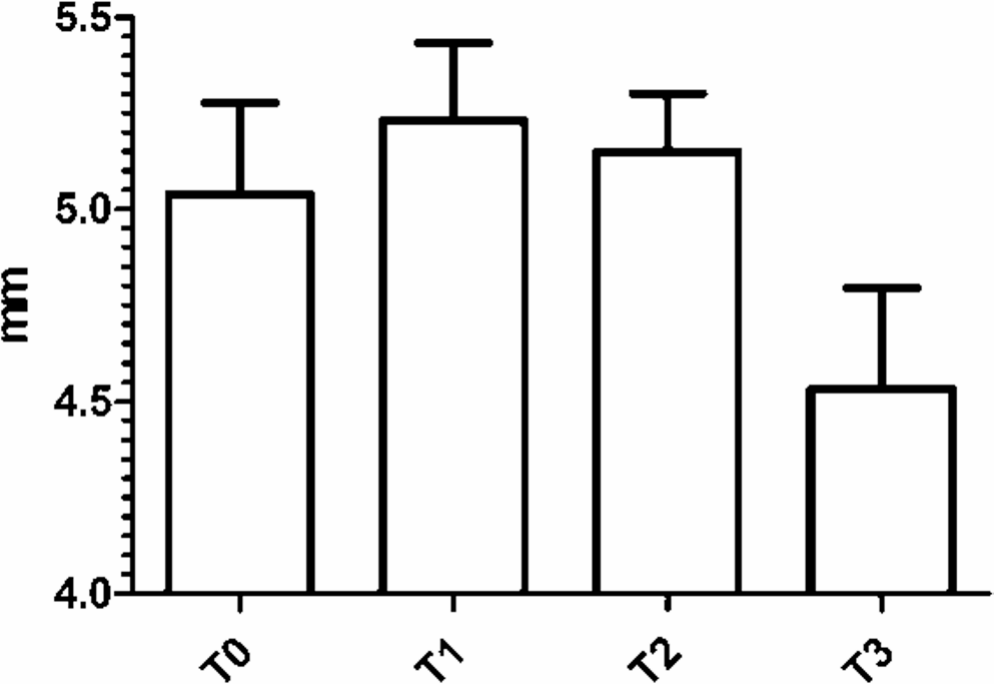
Bar plot showing mean optic nerve sheath diameters (ONSD) assessed through standardized orbital ultrasound (SOUS). Measurements were taken both preoperatively (T0) and postoperatively, at 3 (T1), 6 (T2), and 12 months (T3) after surgery. Acetazolamide dosage was increased after ONSDs were found to be consistently elevated during the second follow-up at 6 months (T2). Error bars express standard deviation (SD)

In 5 out of the 23 (22%) patients who underwent SOUS evaluation, the dosage was increased to 1–2 g/day during follow-up due to the persistence of symptoms (*n* = 4/5, 80%) and/or the persistence of abnormal ONSD values in consecutive follow-up measurements (T1 and T2). In these patients, the mean preoperative (T0) ONSD was 5.0 mm ± 0.2. Three months after surgery (T1), the mean ONSD was 5.2 mm ± 0.2. The mean increase in OUSD between T0 and T1 was 0.2 mm ± 0.3 (*p* = 0.27, 95% CI = − 0.8492 to 0.4692). Six months after surgery (T2), ONSD was found to be 5.2 mm ± 0.2. After 12 months (T3), following the dosage increase, the mean ONSD was 4.5 mm ± 0.3 (Fig. 4). The mean decrease in OUSD between T2 and T3 was 0.7 mm ± 0.3 (*p* = 0.02, 95% CI = − 0.0159 to 0.14090).

Four patients (*n* = 4/48, 8%) required surgical revision due to CSF leak recurrence within the first year. None of them underwent SOUS preoperatively and during follow-up. However, it is essential to note that within this group of patients who underwent ultrasonographic evaluation, only one (*n* = 1/23, 4.5%) experienced a CSF leak recurrence after 2 years, following spontaneous discontinuation of Acetazolamide. In this patient, ONSD was found to be 5.4 mm in the right eye and 5.3 mm in the left eye at T3. Two years later at readmission, ONSD was 4.8 mm in the right eye and 4.7 mm in the left eye. Following the second endoscopic endonasal procedure for CSF leak repair, ONSD was found to be increased again bilaterally (5.4 mm in the right eye, 5.2 mm in the left eye).

## Discussion

This study presents a comprehensive longitudinal analysis of 48 patients who underwent surgical repair for spontaneous cerebrospinal fluid (sCSF) fistulas at Fondazione Policlinico Universitario A. Gemelli IRCCS between October 2003 and January 2023.

The demographic characteristics of our cohort are in line with previous studies on spontaneous sCSF leaks. The median age of our cohort was 53.5 years, and most patients were female (*n* = 32/48, 67%). Most of the patients were overweight, with 23% being obese. These findings suggest a higher incidence of sCSF leaks in middle-aged women with elevated BMI [3]. 

The hallmark symptom in all patients was rhinoliquorrhea, consistent with the typical presentation of sCSF leaks [1, 3, 5, 8]. Headache, nausea, and vomiting were the most common associated symptoms. Pneumococcal meningitis was the initial presentation in 6.5% of our patients (*n* = 3/48), highlighting the risk of noticeable complications in cases of untreated or undiagnosed CSF leaks [48, 49]. 

Our study found that most patients had a single site of leakage, with the ethmoid bone being the most common site (*n* = 29/43, 67.5%), followed by the sphenoid bone (*n* = 13/43, 30%). This distribution is in line with what is typically observed in sCSF leaks, where the ethmoid bone is often the primary site of leakage [1, 3, 5, 8]. Detecting multiple leakage points in 10.5% of cases (*n* = 5/48) highlights the need for a thorough diagnostic evaluation.

Imaging plays a critical role in identifying the site of the CSF leak, with most patients undergoing preoperative CT or MRI scans. In cases where preoperative imaging is unclear, intraoperative fluorescein is often used as an additional tool for precise localization of the fistula [3, 8, 18, 50]. In our cohort of patients, intrathecal injection of fluoresceine was required in only a few cases (*n* = 11/48, 23%) when preoperative imaging did not clearly identify the leakage site.

All patients in our cohort underwent an endoscopic endonasal approach to repair their CSF leaks. The high success rate and safety of this minimally invasive technique are well-documented. Our results support endoscopic endonasal surgery as the gold standard treatment for sCSF leaks [9–15, 17]. However, a combined endoscopic and craniotomic approach was necessary in 3 out of 48 (6%) patients to achieve optimal surgical closure. These data underline the importance of individualized surgical planning based on the fistula location, size, and complexity.

Standardized orbital ultrasound (SOUS) is a fast and easily repeatable technique to indirectly assess increased intracranial pressure (ICP) in sCSF leak patients, especially those who already exhibit radiological features of IIH during diagnosis. Moreover, it is a non-invasive technique, free of significant complications compared to lumbar puncture, which is the gold standard in direct ICP evaluation. Furthermore, OUS is cost-effective, as it can be performed during hospitalization without extending the length of stay or in the outpatient setting, and of course it became of paramount importance in the follow up period allowing us to avoid expensive and time consuming execution of MRI [29, 34–40, 51]. 

The experience of the personnel can influence the accuracy of the measurements obtained; however, as previously shown, novice operators may be efficiently trained in a single training session [38]. 

Our study showed an initial increase in ICP following surgery (Fig. 3), consistent with previous findings by Tilak et al. [38] A possible explanation for this is that, before surgery, the sCSF leak itself contributes to a reduction in ICP, resulting in normal or slightly high ONSD values even in the presence of associated IIH. However, after the initiation of medical treatment, ICP levels decreased, suggesting the efficacy of Acetazolamide in normalizing intracranial pressure (Fig. 4). This phenomenon, whereby the active fistula acts as a continuous CSF drainage route that artificially lowers ICP and may mask underlying intracranial hypertension, is well documented in the IIH-associated sCSF leak literature. Once the fistula is surgically sealed, CSF dynamics normalize and the pre-existing tendency toward intracranial hypertension becomes unmasked, transiently elevating ICP and ONSD in the early postoperative period. Additional contributing factors may include: (a) perioperative surgical manipulation potentially affecting cerebral venous drainage and CSF resorption; (b) the initial 500 mg/day Acetazolamide dose may be insufficient in patients with more pronounced IIH, introducing a therapeutic lag; and (c) individual pharmacological variability in response to Acetazolamide may delay ONSD normalization in a subset of patients.

Achieving this reduction in ICP is a key therapeutic goal, as it is essential to prevent the recurrence of the leakage. Maintaining normalized ICP values post-operatively is crucial for long-term success and avoiding further surgical interventions. The initial dose of Acetazolamide (500 mg daily) can be adjusted upward until stable normal ONDS values are achieved.

In our study, an overall 8% recurrence rate (*n* = 4/48) was observed within the first year, which aligns with reported rates in the literature, ranging from 5% to 17% [5, 17, 48, 49]. None of the patients who received SOUS as part of their follow-up experienced a CSF leak during the study period. One likely contributing factor to this significantly lower recurrence rate is that SOUS allowed for the timely detection of abnormal ICP values. The evidence of no IIH response to Acetazolamide helped guide adjustments to the medical regimen. This individualized approach to medical therapy following CSF leak repair, guided by SOUS monitoring, likely played a key role in preventing recurrence and optimizing patient outcomes.

Regarding the comparison between SOUS and lumbar puncture: lumbar puncture remains the gold standard for direct ICP measurement, but it is invasive, carries procedural risks, and cannot be safely repeated at short intervals for dynamic monitoring. In contrast, SOUS is non-invasive, repeatable, cost-effective, and performable in outpatient settings. Its strong correlation with lumbar puncture opening pressure has been well validated [36, 38, 39]. In the context of post-surgical sCSF leak management requiring months of ICP follow-up, SOUS offers a practical advantage that repeated lumbar puncture cannot provide. Of the 5 patients who required dose escalation, 4 (80%) had persistent symptoms that would have independently triggered a dosing review; however, 1 patient (20%) had dose escalation driven primarily by persistently elevated ONSD in the absence of prominent symptoms, illustrating the added clinical value of SOUS-guided monitoring in oligosymptomatic patients. All 4 CSF leak recurrences within the first postoperative year occurred in the non-SOUS cohort (4/25, 16%), while no recurrences occurred in SOUS-monitored patients (0/23). Although this comparison is confounded by different baseline IIH likelihood between groups, it provides a hypothesis-generating signal in support of SOUS-guided perioperative management, awaiting validation in prospective controlled studies.

In the cohort of patients who were followed up with SOUS, the only CSF recurrence occurred after 2 years, in the controlateral nostril and different site, following the spontaneous discontinuation of medical treatment. In confirmation of our data, ONSD was abnormally high at readmission in this patient and increased again after revision surgery. These data emphasize the importance of long-term follow-up and monitoring of patients with sCSF leaks, particularly those showing early signs of Idiopathic Intracranial Hypertension (IIH), to prevent recurrences and complications. Therefore, we routinely use SOUS in our sCSF leak management algorithm in the preoperative setting and also in the follow-up in order to improve and modulate the medical therapy as depicted in Fig. 1.

## Limits of the present study

The present investigation’s retrospective nature may impact the findings’ robustness. Furthermore, the relatively small sample size limits the generalizability of our findings, and more extensive prospective studies are needed to confirm our results. A complete retrospective data collection could be conducted in a limited cohort of CSF leak patients. Additionally, our data systematically includes only the first year of follow-up. Furthermore, important confounding variables were not systematically controlled. Weight management is central to IIH therapy, as BMI reduction directly lowers ICP; however, serial BMI data at each SOUS follow-up visit were not collected in a standardized manner, precluding formal disentanglement of Acetazolamide effects from weight-related ICP changes. Comorbidities (e.g., obstructive sleep apnea, hypothyroidism) and concomitant medications (e.g., corticosteroids, tetracyclines, antiepileptics) that may independently affect ICP were not systematically controlled as confounders. Additionally, a formal IIH diagnosis requires lumbar puncture opening pressure measurement, which was not systematically performed in every patients in the two cohorts; the MRI findings used to select patients for SOUS evaluation are neither fully sensitive nor specific for elevated ICP, and the true prevalence of raised ICP cannot be precisely established from these data alone.

Finally, SOUS is an operator-dependent technique, which may introduce biases into the results. However, using a single expert operator helps mitigate this limitation inherent to the technique.

## Conclusion

In summary, this study contributes essential data on the management of spontaneous CSF leaks, emphasizing the need for careful postoperative long-term monitoring. Our findings highlight the complex relationship between increased ICP, optic nerve sheath diameter (ONSD) changes detectable by OUS, and the risk of recurrence, underscoring the need for personalized treatment strategies. Standardized orbital ultrasound (SOUS) is a promising, non-invasive technique to indirectly detect ICP variations in patients affected by sCSF leaks undergoing surgical repair. Therefore, this diagnostic tool plays an essential role in minimizing the risk of recurrence by assessing the quality of the surgical repair and supporting clinicians in optimizing medical treatment.

## Data Availability

No datasets were generated or analysed during the current study.

## References

[1] Ommaya AK, Di Chiro G, Baldwin M, Pennybacker JB (1968) Non-traumatic cerebrospinal fluid rhinorrhoea. J Neurol Neurosurg Psychiatry 31(3):214–225. 10.1136/jnnp.31.3.214 5303103 10.1136/jnnp.31.3.214 PMC496347

[2] Schlosser RJ, Wilensky EM, Grady MS, Bolger WE (2003) Elevated intracranial pressures in spontaneous cerebrospinal fluid leaks. Am J Rhinol 17(4):191–195 12962187

[3] Mollan SP, Davies B, Silver NC et al (2018) Idiopathic intracranial hypertension: consensus guidelines on management. J Neurol Neurosurg Psychiatry 89(10):1088–1100. 10.1136/jnnp-2017-317440 29903905 10.1136/jnnp-2017-317440 PMC6166610

[4] Aaron GP, Illing E, Lambertsen Z et al (2017) Enlargement of Meckel’s cave in patients with spontaneous cerebrospinal fluid leaks. Int Forum Allergy Rhinol 7(4):421–424. 10.1002/alr.21891 27918153 10.1002/alr.21891

[5] Seth R, Rajasekaran K, Luong A, Benninger MS, Batra PS (2010) Spontaneous CSF leaks: factors predictive of additional interventions. Laryngoscope 120(11):2141–2146. 10.1002/lary.21151 21113927 10.1002/lary.21151

[6] Allensworth JJ, Rowan NR, Storck KA, Woodworth BA, Schlosser RJ (2019) Endoscopic repair of spontaneous skull base defects decreases the incidence rate of intracranial complications. Int Forum Allergy Rhinol 9(10):1089–1096. 10.1002/alr.22399 31403759 10.1002/alr.22399

[7] Oakley GM, Orlandi RR, Woodworth BA, Batra PS, Alt JA (2016) Management of cerebrospinal fluid rhinorrhea: an evidence-based review with recommendations. Int Forum Allergy Rhinol 6(1):17–24. 10.1002/alr.21627 26370063 10.1002/alr.21627

[8] Georgalas C, Oostra A, Ahmed S et al (2021) International consensus statement: spontaneous cerebrospinal fluid rhinorrhea. Int Forum Allergy Rhinol 11(4):794–803. 10.1002/alr.22704 33099888 10.1002/alr.22704

[9] Chaaban MR, Illing E, Riley KO, Woodworth BA (2014) Spontaneous cerebrospinal fluid leak repair: A five-year prospective evaluation. Laryngoscope 124:70–75. 10.1002/lary.24160 23788232 10.1002/lary.24160

[10] Campbell RG, Farquhar D, Zhao N, Chiu AG, Adappa ND, Palmer JN (2016) Cerebrospinal fluid rhinorrhea secondary to idiopathic intracranial hypertension: long-term outcomes of endoscopic repairs. Am J Rhinol Allergy 30(4):294–300. 10.2500/ajra.2016.30.4319 27456599 10.2500/ajra.2016.30.4319

[11] Paludetti G, Sergi B, Rigante M, Campioni P, Galli J (2004) New techniques and technology to repair cerebrospinal fluid rhinorrhea. Acta Otorhinolaryngol Ital 24(3):130–136 15584583

[12] Lobo BC, Baumanis MM, Nelson RF (2017) Surgical repair of spontaneous cerebrospinal fluid (CSF) leaks: a systematic review. Laryngoscope Investig Otolaryngol 2(5):215–224. 10.1002/lio2.75 29094066 10.1002/lio2.75 PMC5655559

[13] Psaltis AJ, Schlosser RJ, Banks CA, Yawn J, Soler ZM (2012) A systematic review of the endoscopic repair of cerebrospinal fluid leaks. Otolaryngology–Head Neck Surg 147(2):196–203. 10.1177/0194599812451090 10.1177/0194599812451090 22706995

[14] Woodworth BA, Prince A, Chiu AG et al (2008) Spontaneous CSF leaks: a paradigm for definitive repair and management of intracranial hypertension. Otolaryngol Head Neck Surg 138(6):715–720. 10.1016/j.otohns.2008.02.010 18503841 10.1016/j.otohns.2008.02.010

[15] Bedrosian JC, Anand VK, Schwartz TH (2014) The endoscopic endonasal approach to repair of iatrogenic and noniatrogenic cerebrospinal fluid leaks and encephaloceles of the anterior cranial fossa. World Neurosurg 82(6):S86–S94. 10.1016/j.wneu.2014.07.018 25496641 10.1016/j.wneu.2014.07.018

[16] Jakob M, Bertlich M, Eichhorn KW, Thudium M, Bootz F *Reconstruction of the Skull Base in Spontaneous Rhinoliquorrhea Rekonstruktion Der Anterioren Schädelbasis Bei Spontaner Rhinoliquorrhoe*10.3205/iprs000137 PMC6637698 31355127

[17] Galli J, Morelli F, Rigante M, Paludetti G (2021) Management of cerebrospinal fluid leak: the importance of multidisciplinary approach. Acta Otorhinolaryngol Ital 41:S18–S29. 10.14639/0392-100X-suppl.1-41-2021-02 34060517 10.14639/0392-100X-suppl.1-41-2021-02 PMC8172102

[18] Boyter E (2019) Idiopathic intracranial hypertension. JAAPA 32(5):30–35. 10.1097/01.JAA.0000554732.85914.91 30969189 10.1097/01.JAA.0000554732.85914.91

[19] Pérez MA, Bialer OY, Bruce BB, Newman NJ, Biousse V (2013) Primary spontaneous cerebrospinal fluid leaks and idiopathic intracranial hypertension. J Neuroophthalmol 33(4):330–337. 10.1097/WNO.0b013e318299c292 24042170 10.1097/WNO.0b013e318299c292 PMC4040082

[20] Adams AS, Russell PT, Duncavage JA, Chandra RK, Turner JH (2016) Outcomes of endoscopic repair of cerebrospinal fluid rhinorrhea without lumbar drains. Am J Rhinol Allergy 30(6):424–429. 10.2500/ajra.2016.30.4371 28124654 10.2500/ajra.2016.30.4371

[21] Yang Z, Wang B, Wang C, Liu P (2011) Primary spontaneous cerebrospinal fluid rhinorrhea: a symptom of idiopathic intracranial hypertension? J Neurosurg 115(1):165–170. 10.3171/2011.3.JNS101447 21476806 10.3171/2011.3.JNS101447

[22] Friedman DI, Jacobson DM (2002) Diagnostic criteria for idiopathic intracranial hypertension. Neurology 59(10):1492–1495. 10.1212/01.wnl.0000029570.69134.1b 12455560 10.1212/01.wnl.0000029570.69134.1b

[23] Illing E, Schlosser RJ, Palmer JN, Curé J, Fox N, Woodworth BA (2014) Spontaneous sphenoid lateral recess cerebrospinal fluid leaks arise from intracranial hypertension, not Sternberg’s canal. Int Forum Allergy Rhinol 4(3):246–250. 10.1002/alr.21262 24407877 10.1002/alr.21262

[24] Schlosser RJ, Woodworth BA, Wilensky EM, Grady MS, Bolger WE (2006) Spontaneous cerebrospinal fluid leaks: a variant of benign intracranial hypertension. Ann Otol Rhinol Laryngol 115(7):495–500. 10.1177/000348940611500703 16900803 10.1177/000348940611500703

[25] Chaaban MR, Illing E, Riley KO, Woodworth BA (2013) Acetazolamide for high intracranial pressure cerebrospinal fluid leaks. Int Forum Allergy Rhinol 3(9):718–721. 10.1002/alr.21188 23733323 10.1002/alr.21188

[26] Teachey W, Grayson J, Cho DY, Riley KO, Woodworth BA (2017) Intervention for elevated intracranial pressure improves success rate after repair of spontaneous cerebrospinal fluid leaks. Laryngoscope 127(9):2011–2016. 10.1002/lary.26612 28512741 10.1002/lary.26612

[27] Soler ZM, Schlosser RJ (2013) Spontaneous cerebrospinal fluid leak and management of intracranial pressure. Adv Otorhinolaryngol 74:92–103. 10.1159/000342284 23257556 10.1159/000342284

[28] Ossoinig KC (1979) Standardized echography: basic principles, clinical applications, and results. Int Ophthalmol Clin 19(4):127–210 395120

[29] Ransom ER, Palmer JN, Kennedy DW, Chiu AG (2011) Assessing risk/benefit of lumbar drain use for endoscopic skull-base surgery. Int Forum Allergy Rhinol 1(3):173–177. 10.1002/alr.20026 22287368 10.1002/alr.20026

[30] Albu S, Emanuelli E, Trombitas V, Florian IS (2013) Effectiveness of lumbar drains on recurrence rates in endoscopic surgery of cerebrospinal fluid leaks. Am J Rhinol Allergy 27(6):e190–e194. 10.2500/ajra.2013.27.3986 24274213 10.2500/ajra.2013.27.3986

[31] Kristiansson H, Nissborg E, Bartek J, Andresen M, Reinstrup P, Romner B (2013) Measuring elevated intracranial pressure through noninvasive methods: a review of the literature. J Neurosurg Anesthesiol 25(4):372–385. 10.1097/ANA.0b013e31829795ce 23715045 10.1097/ANA.0b013e31829795ce

[32] Amin D, McCormick T, Mailhot T (2015) Elevated intracranial pressure diagnosis with emergency department bedside ocular ultrasound. Case Rep Emerg Med 2015:1–3. 10.1155/2015/385970 10.1155/2015/385970 PMC4637462 26587297

[33] Tamburrelli C, Salgarello T, Caputo CG, Giudiceandrea A, Scullica L (2000) Ultrasonographic evaluation of optic disc swelling: comparison with CSLO in idiopathic intracranial hypertension. Invest Ophthalmol Vis Sci 41(10):2960–2966 10967051

[34] Wang LJ, Chen LM, Chen Y et al (2018) Ultrasonography assessments of optic nerve sheath diameter as a noninvasive and dynamic method of detecting changes in intracranial pressure. JAMA Ophthalmol 136(3):250–256. 10.1001/jamaophthalmol.2017.6560 29392301 10.1001/jamaophthalmol.2017.6560 PMC5885896

[35] Chen LM, Wang LJ, Hu Y, Jiang XH, Wang YZ, Xing YQ (2019) Ultrasonic measurement of optic nerve sheath diameter: a non-invasive surrogate approach for dynamic, real-time evaluation of intracranial pressure. Br J Ophthalmol 103(4):437–441. 10.1136/bjophthalmol-2018-312934 30361274 10.1136/bjophthalmol-2018-312934 PMC6691934

[36] Caffery TS, Perret JN, Musso MW, Jones GN (2014) Optic nerve sheath diameter and lumbar puncture opening pressure in nontrauma patients suspected of elevated intracranial pressure. Am J Emerg Med 32(12):1513–1515. 10.1016/j.ajem.2014.09.014 25284485 10.1016/j.ajem.2014.09.014

[37] Chang HK, Wu JC, Liu L (2018) Measuring optic nerve sheath diameter as a proxy for intracranial pressure. JAMA Ophthalmol 136(11):1310–1311. 10.1001/jamaophthalmol.2018.3435 30177991 10.1001/jamaophthalmol.2018.3435

[38] Tilak AM, Yang LC, Morgan J et al (2023) Optic nerve sheath diameter correlates to intracranial pressure in spontaneous CSF leak patients. Int Forum Allergy Rhinol 13(8):1518–1524. 10.1002/alr.23120 36541893 10.1002/alr.23120

[39] Kimberly HH, Shah S, Marill K, Noble V (2008) Correlation of optic nerve sheath diameter with direct measurement of intracranial pressure. Acad Emerg Med 15(2):201–204. 10.1111/j.1553-2712.2007.00031.x 18275454 10.1111/j.1553-2712.2007.00031.x

[40] Liu D, Li Z, Zhang X et al (2017) Assessment of intracranial pressure with ultrasonographic retrobulbar optic nerve sheath diameter measurement. BMC Neurol. 10.1186/s12883-017-0964-5 28962603 10.1186/s12883-017-0964-5 PMC5622417

[41] Soldatos T, Chatzimichail K, Papathanasiou M, Gouliamos A (2009) Optic nerve sonography: a new window for the non-invasive evaluation of intracranial pressure in brain injury. Emerg Med J 26(9):630–634. 10.1136/emj.2008.058453 19700575 10.1136/emj.2008.058453

[42] Rajajee V, Vanaman M, Fletcher JJ, Jacobs TL (2011) Optic nerve ultrasound for the detection of raised intracranial pressure. Neurocrit Care 15(3):506–515. 10.1007/s12028-011-9606-8 21769456 10.1007/s12028-011-9606-8

[43] Shrestha B, Shrestha P, Ghale P, Lakshmipathy G (2021) Correlation between invasive intracranial pressure monitoring and optic nerve sheath diameter in patients with traumatic brain injury. Kathmandu Univ Med J (KUMJ) 19(74):221–224 34819440

[44] Frassanito P, De Bonis P, Caputo CG et al (2012) Blinding Empty Sella. Arch Neurol 69(7). 10.1001/archneurol.2011.2137 10.1001/archneurol.2011.2137 22409938

[45] Salgarello T, Tamburrelli C, Falsini B, Giudiceandrea A, Colotto A (1996) Optic nerve diameters and perimetric thresholds in idiopathic intracranial hypertension. Br J Ophthalmol 80(6):509–514. 10.1136/bjo.80.6.509 8759260 10.1136/bjo.80.6.509 PMC505521

[46] Haritoglou C, Herzum H, Ehrt O, Ossoinig KC, Kampik A (2002) [Echographic differential diagnosis of optic nerve widening]. Ophthalmologe 99(7):559–565. 10.1007/s00347-001-0573-x 12148304 10.1007/s00347-001-0573-x

[47] World Medical Association Declaration of Helsinki (2024) JAMA Oct. 10.1001/jama.2024.21972

[48] Geltzeiler M, Carrau R, Chandra R et al (2019) Management of spontaneous cerebrospinal fluid leaks. Int Forum Allergy Rhinol 9(3):330–331. 10.1002/alr.22318 30758917 10.1002/alr.22318

[49] Zocchi J, Pietrobon G, Lepera D et al (2021) Spontaneous CSF leaks and IIH: a flawless connection? An experience with 167 patients. Laryngoscope 131(2):E401–E407. 10.1002/lary.28828 32557740 10.1002/lary.28828

[50] Holbrook J, Saindane AM (2017) Imaging of intracranial pressure disorders. Neurosurgery 80(3):341–354. 10.1227/NEU.0000000000001362 27471977 10.1227/NEU.0000000000001362

[51] Qayyum H, Ramlakhan S (2013) Can ocular ultrasound predict intracranial hypertension? A pilot diagnostic accuracy evaluation in a UK emergency department. Eur J Emerg Med 20(2):91–97. 10.1097/MEJ.0b013e32835105c8 22327166 10.1097/MEJ.0b013e32835105c8

